# Mustard Leaf Extract Suppresses Psychological Stress in Chronic Restraint Stress-Subjected Mice by Regulation of Stress Hormone, Neurotransmitters, and Apoptosis

**DOI:** 10.3390/nu12123640

**Published:** 2020-11-26

**Authors:** Kyung-A. Hwang, Hye-Jeong Hwang, Yu Jin Hwang, Young Jun Kim

**Affiliations:** 1 Department of Agrofood Resources, National Institute of Agricultural Sciences, Rural Development Administration, Jeollabuk-do 55365, Korea; hjh1027@korea.kr (H.-J.H.); yjhwang1022@korea.kr (Y.J.H.); 2 Department of Food and Biotechnology, Korea University, Sejong 30019, Korea; yk46@korea.ac.kr

**Keywords:** *Brassica juncea*, mustard leaf, chronic restraint stress, stress hormone, apoptosis, depression, neurotransmitters

## Abstract

Mustard leaf (*Brassica juncea* var. *crispifolia* L. H. *Bailey*) has been reported to have psychological properties such as anti-depressant activities. However, studies on chronic stress and depression caused by restraint have not been conducted. Therefore, this study aimed to evaluate the effects of a mustard leaf (ML) extract on chronic restraint stress (CRS) in mice. Male mice were subjected to a CRS protocol for a period of four weeks to induce stress. The results showed that the ML extract (100 and 500 mg/kg/perorally administered for four weeks) significantly decreased corticosterone levels and increased neurotransmitters levels in stressed mice. Apoptosis by CRS exposure was induced by *Bcl-2* and *Bax* expression regulation and was suppressed by reducing *caspase-3* and *poly (ADP-ribose) polymerase* expression after treatment with the ML extract. Our results confirmed that apoptosis was regulated by increased expression of brain-derived neurotrophic factor (*BDNF*). Additionally, cytokine levels were regulated by the ML extract. In conclusion, our results showed that the ML extract relieved stress effects by regulating hormones and neurotransmitters in CRS mice, *BDNF* expression, and apoptosis in the brain. Thus, it can be suggested that the studied ML extract is an agonist that can help relieve stress and depression.

## 1. Introduction

The modern society is exposed to excessive stress and diseases due to heavy workload, difficult human relationships, and career fatigue. Stress studies, which understand the causes of serious social illness, use chronic restraint stress (CRS) animal models as one of the techniques for monitoring psychological and physiological changes caused by stress. In many studies, the CRS method was confirmed to suppress behavioral and physiological functions that caused the retraction of dendrites i CA3 pyramidal neurons [1,2,3,4]. Dendritic retraction caused by stress is due to the reduction in brain-derived neurotrophic factor (BDNF) synthesis and mRNA expression of hippocampal neurotrophic elements. BDNF is widely distributed in the central nervous system (CNS) and plays an important role in maintaining the dendrites of various CNS neurons [5,6,7]. In particular, the synthesis of BDNF in the hippocampus is regulated by several neurotransmitters and hormones, whose secretion is controlled by external stimuli and stress [8,9]. Neurotransmitters are secreted from the hypothalamus in the CNS and include dopamine (DA), serotonin (5-hydroxytryptamine; 5-HT), and norepinephrine (NE), which regulate physiological activities such as emotional state, heart rate, blood pressure, and increased blood flow to the skeletal system. Glucocorticoids—steroid hormones—are secreted from the adrenal cortex during stress conditions and play a vital role in stress regulation. Additionally, stress not only affects dendrites and hormone secretion in the CNS, but also triggers an inflammatory response in the CNS through the activity of astrocytes. IL-1β is a pro-inflammatory cytokine that plays a key role in CNS inflammation, leading to cellular brain damage by inducing neuronal cell death through the production of immunomodulatory molecules such as arachidonic acid derivatives and nitric oxide (NO). To limit this process, mechanisms for the production of anti- or pro-inflammatory cytokines such as IL-4, IL-10, IFN-γ, TNF-α, and IL-12 are needed [10,11]. However, if a stress condition persists, cardiac diseases, cerebrovascular diseases, sleep disorders, and depression may be caused by a decrease in the synthesis of nerve growth factors in the brain, resulting in an imbalance between neurotransmitters and hormones [12]. Depression is a chronic stress disorder that involves apoptosis mechanisms. The process of apoptosis is achieved through a balance between intracellular *Bcl-2-associated X protein* (*Bax*) and *B-cell lymphoma 2* (*Bcl-2*), while anti-depressants such as fluoxetine regulate their expression [13,14].

The major biomarkers *BDNF, Bcl-2*, and *Bax* are regulated by stress and depression, and *BDNF* exerts a neuroprotective effect by regulating members of the Bcl-2 family [15]. However, when excessive or long-term external stimulation is applied, the Bcl-2 family activates *caspase-3* and *poly (ADP-ribose) polymerase* (*PARP*) as the expression of *BDNF* decreases, and eventually apoptosis occurs, causing brain nerve damage impaired brain function [16].

Recently, many studies on the efficacy and mechanisms of anti-stress and antidepressant products derived from plants have been conducted. Studies have revealed that *Hypericum* species normalize brain serotonin levels, Rosmarinus officinalis and curcumin from *Curcuma longa* increase dopamine production [17]. Besides, an *Erythrina variegata* bark extract regulated the oxidative deamination of monoamines in the brain [18]. In addition to the plants mentioned above, many studies have been reported on the anti-depressant efficacy of various plant extracts [19]. However, few studies have reported the exact mechanism by which mustard leaves (*Brassica juncea* var. *crispifolia* L. H. *Bailey*), of the dicotyledonous belonging to the *Brassicaceae* family [20] improve stress and depression, and studies of this plant have been conducted only on its antidepressant effect [21], separation techniques, and improvement of plant tolerance to abiotic stress [22].

Therefore, in this study, we evaluated the stress improvement efficacy of mustard leaves for the development of stress-relieving substances. This study thus aims to provide possible natural alternatives, with less side effects, to drugs used for stress improvement and depression.

## 2. Materials and Methods

### 2.1. Chemicals and Reagents

Dulbecco’s modified Eagle medium (DMEM) and penicillin/streptomycin antibiotics (P/S) were purchased from Gibco (Gaithersburg, MD, USA); 3-(4,5-dimethylthiazol-2-yl)-2,5-diphenyltetrazolium bromide (MTT) reagent and corticosterone were purchased from Sigma-Aldrich (St. Louis, MO, USA). Serotonin, dopamine, interleukin-12 (IL-12), tumor necrosis factor-α (TNF-α), and interferon-γ (IFN-γ) ELISA kits were procured from Abcam (Cambridge, MA, USA).

### 2.2. Preparation of a Mustard Leaf (ML) Extract

Mustard leaves were purchased from a local market (Chungju-si, Chungcheongbuk-do, Korea). The purchased ML was pulverized and passed through a 40-mesh sieve after hot-air drying at 60 °C for 24 h. ML powder was extracted with four volumes of 70% ethanol, filtered under reduced pressure, and lyophilized using a freeze-dryer at −70 °C.

### 2.3. Animals and Treatments

C57BL/6 mice (6-week-old, male) were purchased from Central Lab. Animal Inc. (Seoul, Korea). The mice were housed under controlled temperature (22 °C ± 2 °C) with a relative humidity of 50% ± 10% and 12 h light/12 h dark cycles. After one week of acclimatization, the mice were randomly divided into five groups (*n* = 5) and treated as shown in Table 1. The ML extract was perorally administered to mice (100 and 500 mg/kg) once daily for 4 weeks, and the positive control group received paroxetine (10 mg/kg) as an anti-depressant. All animal experiments were approved by the Institutional Animal Care and Use Committee of the National Institute of Agricultural Sciences (NAS201904), and all procedures were conducted in accordance with the Animal Experiment Guidelines of the NAS.

### 2.4. Chronic Restraint Stress (CRS) Procedure in Mice

To induce CRS, mice were immobilized in individual restraining chambers (width: 6.3 cm, length: 9 cm) for 2 h per day for 28 consecutive days with suppressed movement [23]. During restraint stress, the animals were not only physically compressed but also deprived of food and water. The normal group also did not receive food and water during the time the CRS groups were subjected to stress. The mice were then anaesthetized and sacrificed to obtain whole brain tissues [24,25] and plasma for biochemical analyses, immediately after the last restraint in the case of CRS.

### 2.5. Stress Hormone Estimation

The amount of corticosterone in mouse plasma was measured using a corticosterone enzyme-linked immunosorbent assay (ELISA) kit (Cusabio Technology LLC., Houston, TX, USA) following the procedure described by the kit manufacturer. After the whole process, the absorbance was measured at 450 nm using a microplate spectrophotometer (SpectraMax M5, Molecular Devices, LLC., Sunnyvale, CA, USA), and the results were expressed as pg/mL.

### 2.6. Neurotransmitter Estimation

The neurotransmitters 5-HT, DA, and NE were estimated from the brain tissue homogenates of mice by ELISA according to the procedure described by the kits’ manufacturer (Cusabio Technology LLC., Houston, TX, USA). To brain obtain tissue homogenates, brain tissue and PBS were placed in a Lysing Matrix D tube (MPBio, Santa Ana, CA, USA), then homogenized using the FastPrep 24-instrument (MPBio, Santa Ana, CA, USA), and centrifuged for 1 min at 10,000 rpm. The obtained supernatants were used in the experiments. The results were expressed as ng/mL and pg/mL.

### 2.7. Real-Time PCR Analysis

To determine stress-related mRNA expression levels, real-time reverse transcription polymerase chain reaction (RT-PCR) was performed using a Rotor-Gene Q real-time thermal cycler (Qiagen, Valencia, CA, USA). Total RNA was extracted from brain tissue according to the manufacturer’s instructions using the RNeasy Mini Plus kit (Qiagen, Valencia, CA, USA). Next, cDNA was synthesized from the isolated total RNA using M-mLV Reverse Transcriptase. Real-time PCR was performed using 2X SYBR Green mix (GenDEPOT, Barker, TX, USA), and gene expression levels were normalized to that of the housekeeping gene *glyceraldehyde 3-phosphate dehydrogenase* expression (*GAPDH*). Primer sequences used for real-time quantitative RT-PCR were as follows: *GADPH*, 5’-CGG AGT CAA CGG ATT TGG TC-3’ (forward), 5’-AGC CTT CTC CAT GGT GGT GA-3’ (reverse); *BDNF*, 5’-AGC GTG AAT GGG CCC AAG GCA-3’ (forward), 5’-TGT GAC CGT CCC GCC CGA CA-3’ (reverse); *Bcl-2*, 5’-CAT GCG ACC TCT GTT TGA-3’ (forward), 5’-GTT TCA TGG TCC ATC CTT G-3’ (reverse); *Bax*, 5’-ACA CCT GAG CTG ACC TTG-3’ (forward), 5’-AGC CCA TGA TGG TTC TGA TC-3’ (reverse); *Caspase-3*, 5’-CCT CAG AGA GAC ATT CAT GG-3’ (forward), 5’-GCA GTA GTC GCC TCT GAA GA-3’ (reverse); *IL-4*, 5’-TAG TTG TCA TCC TGC TCT T-3’ (forward), 5’-CTA CGA GTA ATC CAT TTG C-3’ (reverse); *IL-10*, 5’-TCC CTG GAT CAG ATT TAG AGA-3’ (forward), 5’-CAG CCG GGA AGA CAA TAA C-3’ (reverse); *IL-1β*, 5’-GGA CAG AAT ATC AAC CAA CAA GTG ATA-3’ (forward), 5’-GTG TGC CGT CTT TCA TTA CAC AG-3’ (reverse).

### 2.8. Western Blot Analysis

Brain tissue was homogenized in chilled cell lysis buffer (0.5% NP-40, 0.5% Triton X-100, 0.1% sodium deoxycholate, 50 mM Tris-HCl, 150 mM sodium chloride, and 1 mM EDTA) containing protein inhibitors. The dissolved proteins were left on ice for 40 min and then collected after centrifugation at 12,000× *g* for 20 min at 4 °C. Protein concentration was determined using a bicinchoninic acid assay (BCA) (GenDEPOT, Barker, TX, USA). A quantity of 5–20 μg total protein was loaded onto a 4–20% gel for SDS-PAGE, transferred to a PVDF membrane, blocked with skimmed milk for 1 h, and then incubated overnight with primary antibodies against the following proteins at 4 °C: BDNF, Bcl-2, Bax, Caspase-3, PARP, and β-actin (Abcam, Cambridge, MA, USA). The membranes were then incubated with secondary antibodies for 1 h and developed using an enhanced chemiluminescence kit (Pierce, Rockford, IL, USA) and a ChemiDoc image detector (Bio-Rad, Hercules, CA, USA).

### 2.9. Inflammatory Cytokine Estimation

The levels of inflammatory cytokines (IFN-γ, TNF-α, and IL-12) in the plasma of mice were measured using commercially available ELISA kits (R&D Systems, Minneapolis, MN, USA) according to the manufacturer’s instructions. The absorbance of each inflammatory cytokine was measured at 450 nm using a microplate spectrophotometer (Molecular Devices, LLC., Sunnyvale, CA, USA).

### 2.10. Statistical Analyses

Data were expressed as the mean ± standard error of the mean (SEM). Experimental group comparisons were performed using one-way analysis of variance (ANOVA). When significance (*p* < 0.05) was found, the differences of the mean values were determined with the Duncan’s multiple range test. Statistical analyses were performed using SPSS (v21.0; SPSS Inc., Chicago, IL, USA).

## 3. Results

### 3.1. Effect of CRS and ML Extract on Body Weight

**Figure 1:**
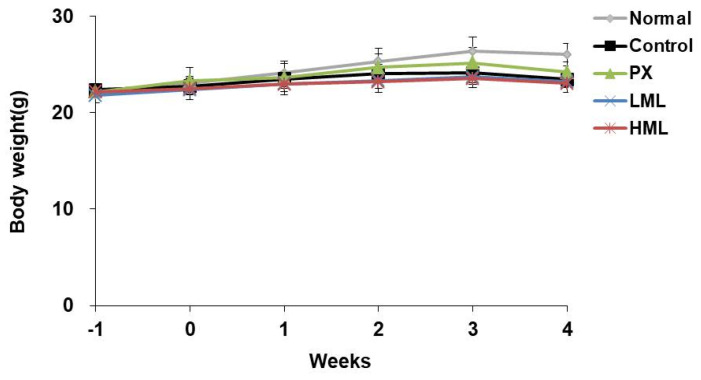
Effect of a mustard leaf (ML) extract on mouse body weight. Data represent means ± SEM (*n* = 5). CRS—chronic restraint stress; PX—paroxetine; LML—low-concentration mustard leaf; HML—high-concentration mustard leaf.

We measured the body weight of mice once a week, and the initial body weight showed no difference between the experimental groups (Figure 1). After CRS exposure for 3 weeks, the stress-induced group showed a significant weight loss compared to the normal group. However, weight loss was not affected by the ML extract.

### 3.2. Effect of the ML Extract on Corticosterone Levels in CRS-Subjected Mice

**Figure 2:**
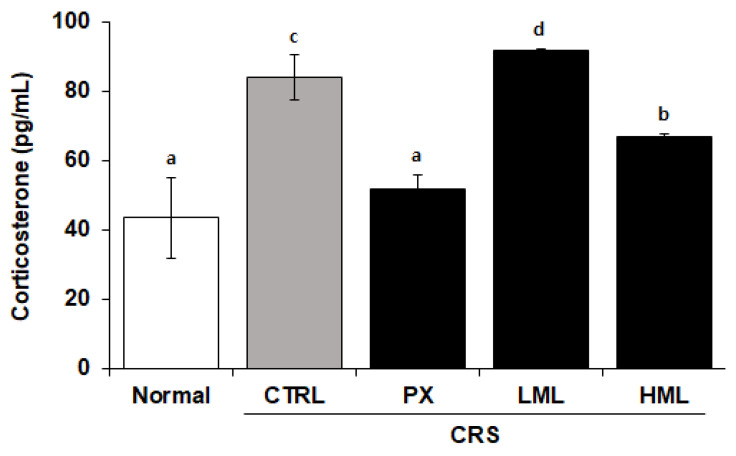
Effect of the ML extracts on corticosterone levels in the plasma. Mice were restrained for 2 h per day for 28 consecutive days after the treatments, and the corticosterone levels were measured in the plasma. Data represent means ± SEM (*n* = 5). Different letters (a–d) above the bars indicate significant differences at *p* < 0.05. CRS—chronic restraint stress; PX—paroxetine; LML—low-concentration mustard leaf; HML—high-concentration mustard leaf.

Corticosterone levels in the plasma were measured to determine the degree of stress exposure. In the normal group, the plasma corticosterone level was 43.7 pg/mL and in the control group, it was 97.2 pg/mL, thereby indicating a 2.2-fold increase compared to the normal group. A paroxetine (PX) group consisting of paroxetine-fed mice and a group treated with a high concentration of ML (HML) extract showed corticosterone levels of 51.8 pg/mL and 32.27 pg/mL, respectively, thus exhibiting a significant decrease compared to the control group (Figure 2).

In a stress situation, corticosterone and cortisol are secreted from the adrenal cortex and break down proteins and fats to raise blood sugar and supply energy to cope with stress [26]. When we measured blood glucose, we observed a tendency to decreased blood glucose in LML and HML groups which, however, was not significant (Supplementary Figure S1).

### 3.3. Effect of the ML Extract on Neurotransmitter Levels in CRS-Subjecetd Mice

**Figure 3:**
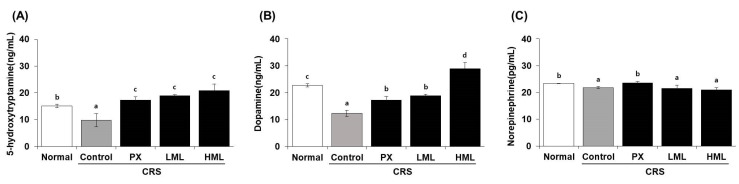
Effect of the ML extracts on monoamines in brain tissue. Mice were restrained for 2 h for 28 consecutive days after the treatments. The neurotransmitters (**A**) 5-HT, (**B**) DA, and (**C**) NE were measured by ELISA. Data represent means ± SEM (*n* = 5). Different letters (a–d) above the bars indicate significant differences at *p* < 0.05. CRS—chronic restraint stress; PX—paroxetine; LML—low-concentration mustard leaf; HML—high-concentration mustard leaf; 5-HT—serotonin; DA—dopamine; NE—norepinephrine.

Neurotransmitters are chemical messengers that regulate many functions and processes in the body. Among them, 5-HT, DA, and NE are fundamental to normal brain function as they are involved in depression and stress. The neurotransmitter levels evaluated in the brain tissue after treatment with the ML extract in CRS-induced mice are shown in Figure 3. The levels of 5-HT and DA in the control CRS group were decreased by 1.5- and 2.0-fold, respectively, compared to the normal, untreated group. However, after the administration of the ML extract, serotonin levels were significantly increased, showing values of 19 and 21 ng/mL after treatment with a low concentration of ML (LML) and a high concentration of HML, respectively, compared to those of the control group, and DA levels were also increased in a dose-dependent manner. These results suggested that the group treated with the ML extract showed a greater recovery tendency than the PX group which was administered paroxetine as an anti-depressant. After CRS, NE levels were reduced in the control group compared to the normal group; however, NE levels were not recovered by treatment with the ML extract.

### 3.4. Effect of the ML Extract on BDNF mRNA Expression in CRS-Subjetced Mice

**Figure 4:**
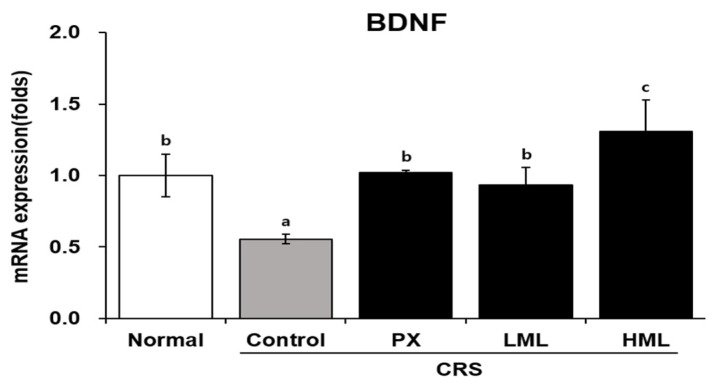
Effect of the ML extracts on *BDNF* mRNA expression in brain tissue. Mice were restrained for 2 h for 28 consecutive days after treatments. *BDNF* mRNA expression was measured by real-time PCR. Data represent means ± SEM (*n* = 5). Different letters (a–c) above the bars indicate significant differences at *p* < 0.05. CRS—chronic restraint stress; PX—paroxetine; LML—low-concentration mustard leaf; HML—high-concentration mustard leaf; BDNF—brain-derived neurotrophic factor.

The synthesis of BDNF has been reported to be regulated by external stimuli and stress, and an increase in the expression of *BDNF* is observed upon antidepressant administration. Therefore, we measured the expression level of *BDNF* mRNA in the brain tissue of CRS-subjected mice (Figure 4). *BDNF* mRNA level was significantly decreased in the control group compared to that in the normal group, while it increased in the ML-treated group depending on the concentration of the ML extract. This experiment showed that HML could increase *BDNF* mRNA levels compared to PX and LML treatments.

### 3.5. Effect of the ML Extract on Apoptosis-Related mRNA Expression in CRS-Subjected Mice

**Figure 5:**
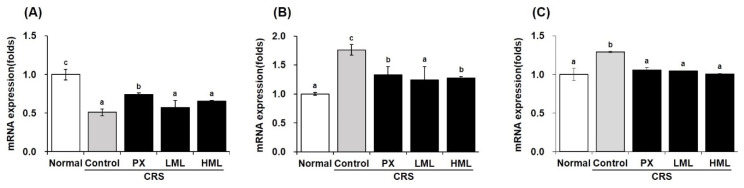
Effects of the ML extracts on mRNA expression of apoptosis-related genes in brain tissue. Apoptosis-related gene were detected by real-time PCR. (**A**) *Bcl-2,* (**B**) *Bax,* (**C**) *Caspase-3.* Data represent means ± SEM (*n* = 5). Different letters (a–c) above the bars indicate significant differences at *p* < 0.05. CRS—chronic restraint stress; PX—paroxetine; LML—low-concentration mustard leaf; HML—high-concentration mustard leaf.

Cell apoptosis occurs in the brain exposed to stress and is known to be an important mechanism underlying depressive behavioral disorders. As the expression of *Bcl-2*, *Bax*, and *Caspase-3* genes regulate death and depression, it was essential to study the mRNA expression levels of these apoptosis-related genes after treatment with the ML extract, which is shown in Figure 5. The stress-subjected control group showed decreased Bcl-2 expression compared to the normal group, while Bcl-2 expression was increased after treatment with the ML extract. Additionally, when the amount of *Bax* and *Caspase-3* expression was increased by stress, the ML extracts significantly decreased their mRNA levels that promoted apoptosis. These results suggest that mustard leaves may help improve stress and depression by controlling the expression of stress-induced cell apoptosis-related gene.

### 3.6. Effect of the ML Extract on Apoptosis-Related Protein Expression in CRS-Induced Mice

**Figure 6:**
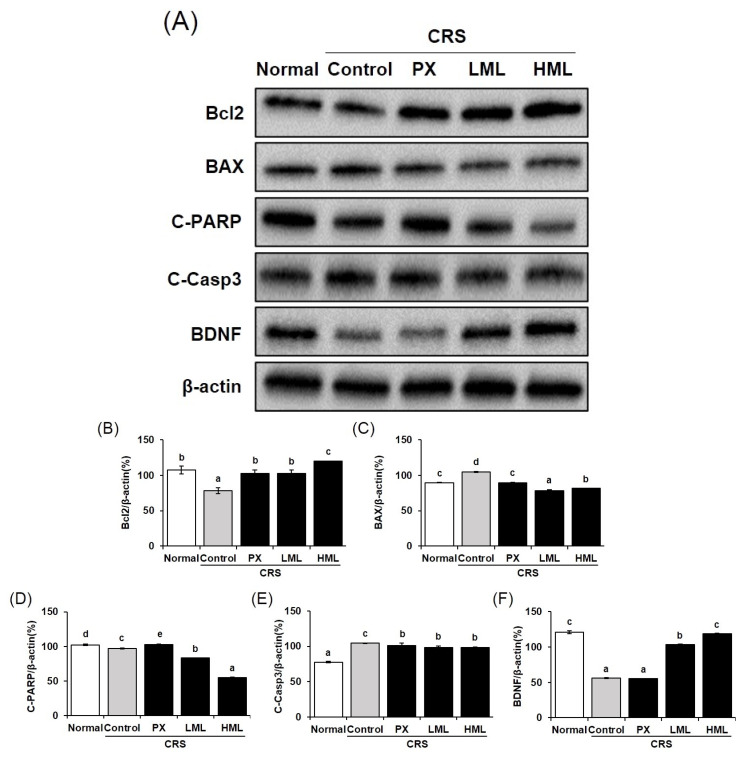
Effects of the ML extracts on the expression of *BDNF* and apoptosis-related proteins in brain tissue. (**A**) *BDNF* and apoptosis-related proteins were detected by Western blot. (**B**–**F**) densitometry analysis of proteins. Different letters (a–e) above the bars indicate significant differences at *p* < 0.05.CRS—chronic restraint stress; PX, paroxetine; LML—low-concentration mustard leaf; HML—high-concentration mustard leaf; C-PARP—Cleaved-PARP; C-Casp3—Cleaved Caspase3.

To elucidate the molecular mechanisms involved in stress-induced apoptosis after treatment with the ML extract, the expression of apoptosis-related proteins was measured. As shown in Figure 6, compared to the control group, the protein expression of *Bax* and cleaved *Caspase-3* in the ML-treated group significantly decreased, while that of *Bcl-2* increased. Additionally, cleaved *PARP* activity was lower in the ML-treated group than in the control group. These data are similar to the results of a study reported by Xia et al. [27] that showed that 2-(4-Methoxyphenyl)ethyl-2-acetamido-2-deoxy-β-D-pyranoside inhibits cell death by reducing cleaved *PARP* expression after inhibiting cleaved *caspase-3* activity during severe DNA damage. Based on a study reporting *BDNF*-mediated regulation of apoptosis via the control of *Bcl-2* and *Bax* expression [28], it can be suggested that by increasing the expression of *BDNF*, ML extracts may suppress apoptosis through regulation of *Bcl-2* and *Bax* expression.

### 3.7. Effect of the ML Extract on Anti- and Pro-Inflammatory Cytokine mRNA Expression in CRS-Subjected Mice

**Figure 7:**
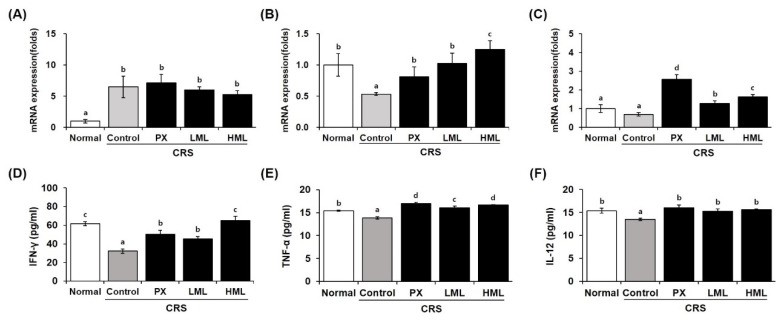
Effect of the ML extracts on anti- and pro-inflammatory cytokine mRNA expression and production in the brain. Mice were restrained for 2 h for 28 consecutive days after the treatments. (**A**) IL-1β, (**B**) IL-10, (**C**) IL-4, (**D**) IFN-γ, (**E**) TNF-α, (**F**) IL-12. (**A**–**C**) Cytokine mRNA expression was detected by real-time PCR. (**D**–**F**) Cytokine production was detected by ELISA kit. Data represent means ± SEM (*n* = 5). Different letters (a–d) above the bars indicate significant differences at *p* < 0.05. CRS—chronic restraint stress; PX—paroxetine; LML—low-concentration mustard leaf; HML—high-concentration mustard leaf.

Brain damage stimulated by stress is controlled by pro- and anti-inflammatory cytokines. Figure 7 shows the expression of cytokines regulated by ML extracts. The control group subjected to CRS showed increased expression of IL-1β mRNA, a pro-inflammatory cytokine; we confirmed that it was decreased in a dose-dependent manner by ML extracts. On the contrary, the mRNA expression of the anti-inflammatory cytokines *IL-4* and *IL-10* tended to be reduced by stress in the control group, while it was significantly increased by the ML extracts.

In previous studies, glucocorticoids inhibited the production of TNF-α, IFN-γ, and IL-12 in vitro and in vivo. Catecholamines are also potent inhibitors of natural killer (NK) cell activity. They inhibit the production of IL-12 and IFN-γ, which are essential for NK cell activity. NK cells are known as the most “sensitive” cells to the inhibitory effect of stress, and hence, NK cell activity has been used as a stress-induced immunosuppression index in several studies [29]. Among cytokines, IFN-γ production decreased significantly in the control group, whereas IFN-γ production was significantly increased in the ML-treated group (Figure 7). Mustard leaves also tended to increase TNF-α and IL-12 levels. These cytokines play an important role in immunity regulation. Thus, the ML extract normalized the levels of cytokines inhibited by stress-releasing substances, suggesting that the mustard leaves may also help in immune regulation. Therefore, it is expected that ML extracts regulate brain damage and inflammatory responses caused by stress by regulating cytokines.

## 4. Discussion

The results of the present study showed that four weeks of CRS in mice induced a stress-like state, which could be relieved by the ML extract. These specific effects were accompanied by an increase in serotonin and dopamine and decreased corticosterone levels. They were also associated with the downregulation of *Bcl-2* mRNA and the upregulation of *Bax* mRNA expression in the brain. Moreover, chronic treatment with ML extracts normalized the alterations in hormone and mRNA levels elicited by the stressful condition. It is well established that chronic stress is an important risk factor for mental illnesses such as major depressive disorder and anxiety. Studies in animals have shown that chronic stress models can present behavioral changes that resemble clinical depression, such as locomotor activity deficit, reduced sucrose consumption, and decreased responsiveness to rewarding stimuli [30].

The effective role of monoamines in the pathophysiology of depressive disorders has been extensively investigated, and it is seen that depletion of monoamine neurotransmitters can lead to such disorders. The monoamine hypothesis has demonstrated that 5-HT and DA are important neurotransmitters involved in the etiology of depression. In the current study, neurotransmitters, including 5-HT and DA, were evaluated in the brain of mice, and it was found that these neurotransmitters were suppressed by restraint stress exposure. However, treatment with mustard leaves increased the levels of these neurotransmitters. The findings of this study are in line with those of previous studies [31], wherein monoamine neurotransmitter levels were increased by using anti-depressant drugs.

BDNF, a member of the neurotrophin family, plays critical roles in cell differentiation, neuronal survival, migration, and synaptic plasticity and has been suggested to be involved in the pathogenesis of depression. For instance, decreased plasma levels of BDNF were found in patients with depression [32]. Moreover, the mRNA levels of *BDNF* appeared suppressed in postmortem brains of depressed patients [33]. *BDNF* expression was also reduced in the hippocampus and prefrontal cortex (PFC) in chronic stress studies [1,34]. Increased BDNF levels were found in postmortem brain samples of depressed patients treated with anti-depressants compared with those of non-treated depressed patients [35,36]. Infusion of BDNF into the hippocampus and midbrain area produced anti-depressant-like behavioral effects in rats [37]. In accordance with these observations, we found that chronic ML treatment reversed the reduction of BDNF levels in the brain that was produced by the stress protocol in the current study. In addition to the reduction of BDNF in the presence of stress and depression symptoms, it was observed that the expression levels of pro- and anti-inflammatory cytokines in brain tissue were also regulated [38]. As the mRNA expression of the *IL-1β* gene increased in the brain tissue of the CRS group, the levels of the anti-inflammatory cytokines *IL-4* and *IL-10* decreased. It was expected that the mRNA expression of *IL-4* and *IL-10* would be restored dose-dependently by the ML extracts with the reduction of *IL-1β*, improving stress and suppressing brain tissue damage. These results are similar to those of previous studies in which *IL-1β*, which is produced in astrocytes when the brain is injured, is reduced by regulation of *IL-4* and *IL-10* expression [11]. In addition, it was reported that IL-4 and IL-10 inhibit inflammation and brain damage by inhibiting NF-κB activity in astrocytes [39]. Likewise, it is thought that the production of TNF-α, IFN-γ, and IL-12 is also increased, so that inflammation caused by stress is regulated by the ML extracts, protecting against brain damage. When cells are exposed to stress caused by external stimuli such as DNA damage and oxidative stress, their physiological balance is disrupted, and the cells exhibit a survival response. However, prolonged exposure to stress does not activate the survival response, and eventually apoptosis progresses. The major members of the Bcl-2 family are anti- and pro-apoptotic, *Bcl-2* and *Bax*, which promote or inhibit apoptosis by regulating caspase activity [40,41]. If protein degradation occurs due to *caspase-3* activity, functions such as DNA repair and genomic stability are lost due to structural changes of PARP, and finally, apoptosis occurs [42]. In Yun et al. [43] study, chronic stress inhibited *Bcl-2* mRNA expression and promoted *Bax* mRNA expression, demonstrating the association between stress and apoptosis factors. In addition, in a previous study [44], it was confirmed that stress-induced apoptosis was suppressed by reducing the increase of cleaved *caspase-3* and PARP activity induced by stress. Similarly, in this study, it was confirmed that blue mustard leaves are effective in inhibiting apoptosis by inhibiting cleaved *caspase-3* and PARP activities through the regulation of apoptosis factors of the Bcl-2 family is not activated by stress.

Apoptosis and brain nerve damage induced by CRS are due to the compounds contained in the mustard leaf extract. Mustard leaves contain vinyl propionate, butyl formate, 2-methoxy-4-vinyl phenol, methyl isoeugenol 1, and 3-isopropoxy-5-ethyl phenol compounds [45]. Among these compounds, methyl isoeugenol 1 is known to regulate apoptosis and have anti-cancer efficacy against various cancers [46]. In addition, long-term stress exposure causes neurodegenerative and mental illness by destroying the blood–brain barrier (BBB) and causing the death of brain neurons. Mahmoud et al. reported that eugenol intake in rats after induced aluminum toxicity showed a neuroprotective effect by increasing the activity of BDNF in the brain. Eugenol seems to cross the BBB and acts in the brain by inhibiting the production of lipid peroxidation, and strengthening endogenous antioxidant mechanisms [47]. Garabadu et al. [48] reported the results of a study on the anti-stress efficacy of eugenol through the control of the monoamine system. Therefore, it is estimated that the efficacy of eugenol, among a number of compounds contained in mustard leaves, in inhibiting neuronal damage and apoptosis induced by CRS would be dominant. Chronic stress is a risk factor for depression and causes physiological and behavioral changes in the body. Behavioral changes are closely related to brain neural factors, and phenomena such as loss of PFC, decrease in the number and size of neurons, and decrease in the expression of BDNF were observed in clinical studies of depressed patients [49]. In addition, behavioral changes indicative of depression were confirmed in animal models through behavioral observations in the forced swim test, the learned helplessness test, and the tail-suspension test [50]. Our data support the need for a further in-depth study of the effects of mustard leaves on depression induced by stress.

In conclusion, our results indicate that restraint stress impairs the apoptosis defense system in the brain. Furthermore, mustard leaves decreased both corticosterone and monoamine levels and inhibited apoptosis by controlling the expression of key factors that regulate it. The effects of the ML extract could be linked to the improvement of apoptosis in the brain, suggesting that ML extracts might function as an anti-stress agent.

## Figures and Tables

**Table 1:** Animal experimental groups and treatments.

Group	Treatment (mg/kg)	Condition
Normal	PBS	No CRS
Control	PBS	CRS
PX	Paroxetine (10)	CRS
LML	Low-concentration mustard leaf (100)	CRS
HML	High-concentration mustard leaf (500)	CRS

CRS—chronic restraint stress, PBS—phosphate-buffered saline, PX—paroxetine; LML—low-concentration mustard leaf; HML—high-concentration mustard leaf.

## Supplementary Materials

The following are available online at https://www.mdpi.com/2072-6643/12/12/3640/s1, Figure S1: Effect of ML extract on blood glucose levels.
