# Supplementary material for: Mustard Leaf Extract Suppresses Psychological Stress in Chronic Restraint Stress-Subjected Mice by Regulation of Stress Hormone, Neurotransmitters, and Apoptosis
Source: Nutrients. 2020 Nov 26;12(12):3640. doi: 10.3390/nu12123640 (PMC7760211; doi:10.3390/nu12123640)
Supplement: Supplementary file 1 [file nutrients-12-03640-s001.pdf]

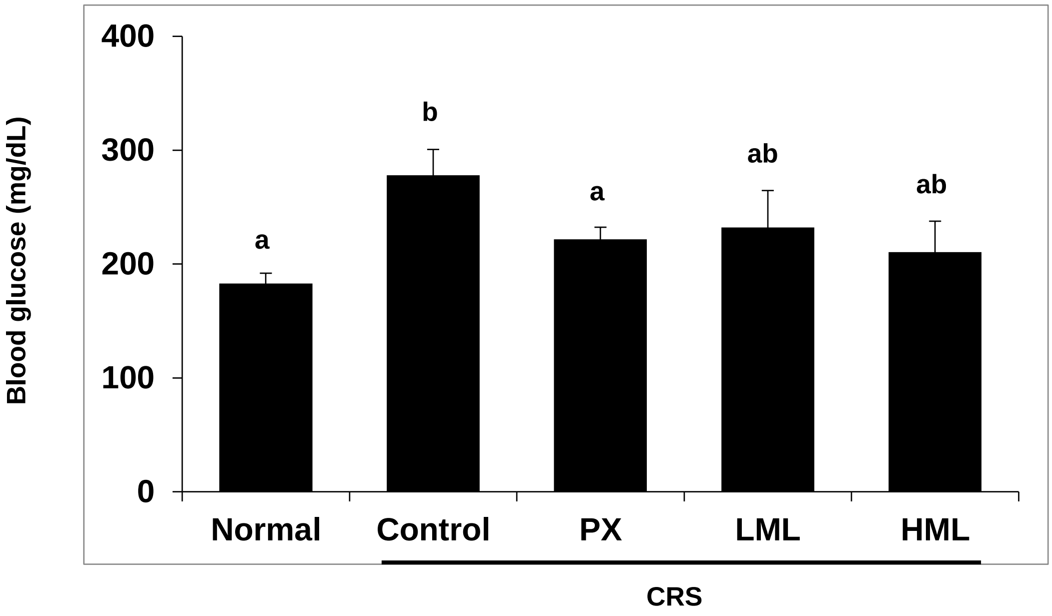

Supplementary Figure S1. Effect of ML extracts on blood glucose levels. Mice were restrained for 2 h per day for 28 consecutive days after the treatments, and the glucose levels were measured in whole blood. Data represent means ± SEM (n = 5). Different letters (a–b) above the bars indicate significant differences at $p < 0.05$. CRS—chronic restraint stress; PX—paroxetine; LML—low-concentration mustard leaf; HML—high-concentration mustard leaf.
